# Utilisation of Paunch Waste as a Natural Fibre in Biocomposites

**DOI:** 10.3390/polym14183704

**Published:** 2022-09-06

**Authors:** Clement Matthew Chan, Darren Martin, Emilie Gauthier, Paul Jensen, Bronwyn Laycock, Steven Pratt

**Affiliations:** 1 School of Chemical Engineering, The University of Queensland, St. Lucia, QLD 4072, Australia; 2 Australian Centre for Water and Environmental Biotechnology, The University of Queensland, St. Lucia, QLD 4072, Australia

**Keywords:** agricultural waste, paunch, natural fibre-polymer composites, biodegradable, biopolymer, biocomposite, extrusion

## Abstract

Paunch is a fibrous solid residue consisting of partially digested feed from the stomachs of processed cattle. It is the largest untapped solid waste stream from animals at meat processing plants, and potentially a valuable source of fibres for the production of sustainable and potentially higher-value natural biocomposite materials. Paunch was obtained from the waste effluent of a red meat processing plant, and the fibre characteristics of the as-obtained material were studied and benchmarked against wood flour and ground buffel grass, with a view to evaluating the potential of paunch as a fibre for polymer composites. The ground paunch possessed a rough fibrous surface and fibre-like characteristics that were comparable to both wood flour and ground buffel grass, demonstrating their potential for use in composites. Without any pre-treatment or compatibilisation, composites of a representative biopolymer, poly(3-hydroxybutyrate-co-3-hydroxyvalerate) (PHBV) and ground paunch were successfully produced for the first time via extrusion, with up to 50 wt% paunch content. Mechanical property analysis showed that, at 30 wt% content, PHBV/ground paunch composites yielded mechanical properties that were comparable to those of composites with ground buffel grass.

## 1. Introduction

Concerns over the environmental issues associated with waste and resource scarcity have raised interest in the utilisation of waste materials in manufacturing processes. Therefore, there is a growing interest in utilising materials from recycled and renewable sources [1], particularly organic waste in general and, more specifically, residues from agricultural and industrial processes, as they are abundantly available at low cost. One of the major residues associated with agro-industries is residual fibre, which has been proven to be a valuable resource for the production of natural fibre–polymer composites and as structural materials [2,3]. These residual fibres can be potentially a more-sustainable substitute for refined/commercial fibres from wood, hemp, etc., which could also offer the same level of reinforcement with a lower cost. For example, composites of high-density polyethylene (HDPE) with 65 wt% of agro-residues of wheat straw, cornstalk and corncob have been successfully produced using extrusion [4]. Amongst these, wheat straw composites showed the best mechanical properties, which was attributed to the hydrocarbon rich surface (lower O/C ratio) of the fibre, which is thus more compatible with the hydrophobic HDPE matrix. Other residue fibres such as carpet waste jute yarns, sunflower stalks, bagasse and soy stalks have also been successfully blended with matrices including polypropylene and epoxy resin [5,6,7].

In this paper we consider the use of paunch, a solid waste stream from meat processing industry, as a fibre for composites. Paunch is a fibrous solid residue consisting of partially digested feed from the stomachs of processed cattle, and potentially biological solids from wastewater treatment [4]. It has a high moisture content, nutrient load and microbial content. The composition includes relatively undigested feed to material close to manure, whereas manure is defined as the organic matters from animal faeces. Up to 40–50% of excreted solid wastes from dairy consist of under-utilised fibrous matter that has been only partially digested by the cow [8]. Paunch is one of the only residues from animal processing that is not sold directly as a product or co-product. It is also one of the largest solid waste streams in Australia, averaging at 30–40 kg per head of cattle; management of paunch incurs significant cost ($20–40/m^3^) [9]. Paunch waste is typically disposed via either composting or landfill which incur resource employment and/or disposal fees [10]. It is obviously an untapped low-cost fibrous material from waste which shows potential for composite applications.

Wastes from animal processing are fibre-rich, but only a few attempts have been made to utilise animal wastes, predominantly on manure and none on paunch to the best of our knowledge, for composite applications. A potential issue with these fibres is that they are not well defined, and the batch-to-batch and facility-to-facility variations of the paunch waste stream are also known to be significant [11], which could affect the resulting composite properties [12]. Still, composites with dried dairy and swine manure with HDPE and high-density polypropylene (HDPP) have been successfully produced with up to 50 wt% fibre content [13]. Dairy manure-filled HDPE has a higher modulus of elasticity and modulus of rupture compared to swine manure-filled HDPE, due to the higher level of fibre reinforcement provided by the small fraction of higher aspect ratio straw fibres in the dairy manure. The potential of anaerobically digested bovine biofibre (ADBF) for manufacturing dry-formed medium-density fibreboards and particleboards using urea formaldehyde as the resin was also explored [14]. Results indicated that particleboards with up to 67 wt% of ADBF (with 33 wt% hammermilled pine wood fibre) met the performance criteria described in ANSI commercial standards, demonstrating a potential market for these waste fibres. A commercial process to make ADBF particleboards has now been developed in a dairy plant in Connecticut, USA, to manufacture biodegradable plant pots [10].

The potential to use paunch as a substitute for virgin natural fibres in composite applications has been underexplored, and the fibre properties from this waste source are not well-characterised. What is more, the waste fibre composites are produced using non-degradable resins, they do not readily biodegrade and are also very challenging to recycle. For this reason, biocomposites made using bio-based and compostable/biodegradable polymer resins are of increasing interest, since the final product is 100% bio-based and compostable/biodegradable. Polylactic acid (PLA) is a stand-out representative among the bio-based compostable polymers and starch-based polymers and polyhydroxyalkanoate (PHA) are among the bio-based biodegradable polymers [15]. Polyhydroxyalkanoate (PHA) is one of the growing ones recently as they can be synthesised intracellularly by bacteria and archaea from organic carbon and are truly biodegradable under ambient conditions (in soil, riverine and marine environments) [16]. The most common PHAs include poly(3-hydroxybutyrate) (PHB) and poly(3-hydroxybutyrate-co-3-hydroxyvalerate) (PHBV). Such PHAs are attractive as composite matrices, because, in addition to their environmental benefits, they have been shown to have useful mechanical properties and are easy to process as a drop-in replacement, with a low melt viscosity, which can be an advantage in the processing of composites [17,18]. PHAs have already shown promises as the polymer matrix in wood–plastic composites [19], as well as waste fibres such as waste wood sawdust and recycled cellulose fibres [20,21]. The field of composites of biopolymers and natural fibres has been growing rapidly recently, and their high costs have been highlighted as one of the major limitations [22]. However, utilising waste fibres as a cost competitive measured has yet to be explored. 

This paper considers paunch as a valuable untapped source of fibres for the production of sustainable and potentially high-performance natural biocomposite materials with PHBV as the polymer matrix. The objective of this study is to explore the potential of paunch as a negative-cost reinforcement/filler for the relatively expensive PHBV, and the same for other biopolymers. A feature of the work is thorough characterisation of the waste fibres, which is lacking in the literature for this sort of residue. Composites of commercially available PHBV and untreated paunch collected from a meat processing facility at increasing loading at 10 wt% increment until the loading limit were produced using extrusion. The fibre properties were characterised and compared to fibres from lignocellulosic material (wood flour) and grass (Cenchrus ciliaris, also called buffel grass) as benchmark, with the former as an established cheap fibre for biocomposites and the latter as a representative of native grasses which have been used as feed for cattle and lambs [19]. The effects of the paunch loading on the mechanical and thermal properties were investigated. PHBV/wood flour and PHBV/ground buffel grass were also fabricated using the same processing method as benchmarks.

## 2. Materials and Methods

### 2.1. Materials

Paunch was collected as a wet fibrous solid (70% moisture content) containing short digested lignocellulosic fibres from cattle at a red meat processor located in Southeast Queensland, Australia from the pile after separation from the red stream (blood and offcuts) and pressing to remove water. The primary source of the paunch is a mixture of digested grass that remained inside the cattle when processed at the facility. The as-collected paunch was washed twice by suspending the fibres in water followed by filtering through a nylon mesh filter bag and was then stored in the fridge below 4 °C until drying. The paunch fibres were then dried in a conventional oven at 80 °C over 3 days and then stored until use. 

Radiata pine wood flour (WF) was obtained from Micromilling, Australia. The wood flour was produced by hammer-milling debarked wood logs and sieved to below 300 µm in particle size. Buffel grass (*Cenchrus ciliaris*) was collected from hummocks of buffel grass growing around Deep Well Station, Northern Territory, Australia (23.952° S, 133.917° E). The roots were cut out and the whole grass was washed with warm water and dried under ambient conditions for 3 days. 

Poly(3-hydroxybutyrate-co-3-hydroxyvalerate) (PHBV) was supplied by TianAn Biopolymer, China under the tradename of ENMAT Y1010 and dried in a vacuum oven at 60 °C at −80 kPa for at least 24 h, then stored until use.

### 2.2. Composite Processing

#### 2.2.1. Fibre Preparation

**Figure 1:**
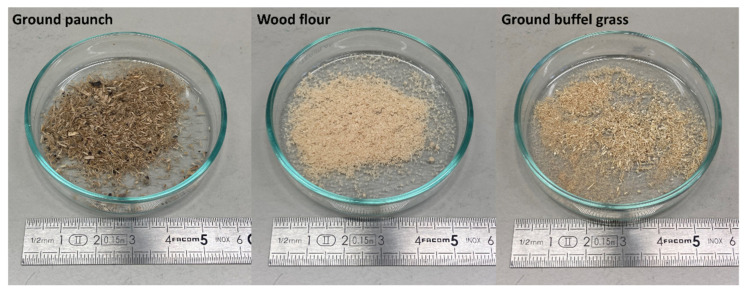
Appearance of ground paunch (**left**), wood flour (**middle**) and ground buffel grass (**right**) after drying.

The composite materials were prepared using a two-step process, consisting of dry mixing followed by melt compounding. Wood flour was used as-is and dried in a vacuum oven for at least 24 h at 80 °C at −80 kPa gauge. The pre-dried paunch and as-received buffel grass were ground using a 2.9 L cutter for 2 min (Robot Coupe Blixer^®^ 2). The ground paunch and buffel grass were then sieved to below 2000 μm in particle size to remove large rocks or particles and further dried in a vacuum oven at 80 °C for at least 24 h at −80 kPa gauge. Figure 1 shows the appearance of the fibres after grinding and drying. The dried fibres were then stored in a vacuum bag until dry mixed, with fibre loadings in the mixture ranging from 0 wt% to 50 wt%, at 10 wt% increments. 

#### 2.2.2. Extrusion Process

A co-rotating twin screw extruder with a diameter of 16 mm and a length-to-diameter ratio of 40:1 was used for melt compounding of the dry mixed blends. A decreasing temperature profile with a maximum barrel temperature of 180 °C and a die temperature of 160 °C was implemented. The screw speed was maintained at 100 rpm. The screw configuration was chosen to consist of kneading elements to provide moderate mixing. A slit die with a cross section dimension of 12 × 2 mm was placed at the die to yield sections of rectangular specimens for mechanical testing. Prior to any characterisation, the samples were first conditioned for at least 2 weeks at a controlled temperature and humidity of 25 °C and 50%, respectively, to allow for slow (secondary) crystallisation and equilibrium moisture content. 

### 2.3. Characterisation of Fibre Properties

#### 2.3.1. Fibre Dimensions Analysis

A Morfi Neo fibre analyser was used to characterise the distribution of fibre length and width. All fibres were dispersed in water to make a solid content of approximately 0.5 g/L and were passed through a tight window. The fibre dimensions were obtained through automatic image processing. Three replicate runs were performed to account for variations. 

#### 2.3.2. Acid Detergent Fibre (ADF) and Neutral Detergent Fibre (NDF) Analysis

The cellulose, hemicellulose and lignin contents of the fibres were determined by the combination of ADF and NDF analysis. NDF and ADF concentrations were measured in 0.45–0.50 g samples with an ANKOM200 Fibre Analyser (Model 200, ANKOM Technology, Macedon, NY, USA) filter bag technique [23]. Neutral detergent or acid detergent solutions, and for NDF also 38 ANKOM heat stable alpha-amylase (ANKOM Technology FAA, Macedon, NY, USA), were added to the analyser vessel and agitated for 75 min (NDF) or 60 min (ADF). Bags were then washed with tap water (90–95 °C) repeatedly, and for ADF until the washings were neutral in pH. After the final rinse the bags were squeezed to remove excess water and then soaked in acetone for 5 min before drying.

#### 2.3.3. Fourier Transform Infrared Spectroscopy (FT-IR)

The surface functional group of the fibre samples was identified using a Thermo Scientific Nicolet iS50 Fourier transform infrared spectrometer using reflection mode and equipped with an Attenuated total reflection (ATR) diamond module. Fibres were dried in a vacuum oven for at least 24 h at 80 °C at −80 kPa gauge before analysis. Two spectra were collected between the ranges of 4000–400 cm^−1^ at a resolution of 4 cm^−1^ and with 128 scans per run. 

### 2.4. Characterisation of Extruded Composites

#### 2.4.1. Gel Permeation Chromatography (GPC)

Gel permeation chromatography (GPC) was used to determine the molecular weight of PHA. Samples were dissolved at a concentration of 2.5 mg/mL in HPLC grade chloroform in capped glass tubes on a heating block at 75 °C for 30 min. An Agilent 1260 Infinity Multi Detector Suite system (Cheshire, UK) fitted with a column set of MW range of 0.5 to 1700 kDa was used. The columns were kept at 30 °C. A refractometer, at 30 °C, was used to detect the signals. A chloroform flow rate of 1 mL/min was maintained for the analysis. Narrowly distributed molecular weight polystyrene standards were used for calibration and the Mark-Houwink equation was used for molecular weight calculations. The Mark-Houwink constants for polystyrene (K = $7.2\times 10^{-5}$ dL/g and α = 0.76) and for P(3HB) in chloroform (K = $7.7\times 10^{-5}$ dL/g and α = 0.82) were taken from the literature [24,25]. Three replicates were performed to verify the reproducibility of the results, where the average is reported. 

#### 2.4.2. Mechanical Testing

Tensile tests were performed according to ASTM D638 standard on an Intron 5584 with a 1 kN electronic load cell. Sections of extruded rectangular strips were laser cut into Type V dogbone-shaped specimen. Tests were performed at a rate of 1 mm/min until fracture. The extensiometer AVE2 system was used to obtain an accurate strain value across the narrow region of the specimens. Ten replicates were performed on each sample. 

#### 2.4.3. Differential Scanning Calorimetry (DSC)

A differential scanning calorimeter Q2000 (TA Instruments) under a constant nitrogen flow of 50 mL/min was used to determine the thermal properties of the PHA matrix. Samples of 2.0 to 4.0 mg were placed in a sealed aluminium pan and were analysed using standard DSC heating and cooling scans. Each sample was heated from 25 °C to 185 °C at 10 °C/min and kept isothermal for 0.1 min, and then cooled to −70 °C at 10 °C/min. The melting temperature, $T_{m}$, and enthalpy of fusion, $\Delta H_{m}$, were determined from the heating cycle. The melt crystallisation temperature, $T_{mc}$, and enthalpy of melt crystallisation, $\Delta H_{mc}$, were determined from the cooling cycle. Two replicates were performed to verify the reproducibility of the results, where the average is reported. 

#### 2.4.4. Scanning Electron Microscopy (SEM)

Samples were assessed using a Hitachi SU3500 scanning electron microscope under secondary electron mode. Fibre samples were prepared by spreading them onto the sample holder. Composites samples were prepared by freezing the specimen before snapping to expose the fractured surface. All samples were coated with ~30 nm of Iridium and vacuum dried at room temperature for at least 24 h before imaging. Image acquisition was done on the Iridium-coated samples at 5.00 kV accelerating voltage and approximately 10 mm working distance.

## 3. Results and Discussion

### 3.1. Fibre Chemistry

The neutral detergent fibre (NDF) content and the individual cellulose, hemicellulose and lignin contents of the ground paunch are presented in comparison with wood flour and ground buffel grass (Table 1). It can be seen that the paunch had a composition that was more similar to buffel grass than to wood, which is not surprising given that the cattle were predominantly fed with grass rather than woody feedstocks. When benchmarking against the chemistry data of natural fibres in the literature, the cellulose content of paunch is lower than the fibres from plants such as flax, hemp or jute, whereas the lignin content is lower than that from wood and straw [22,26]. The chemistry of paunch is more comparable to grass, which has a high hemicellulose content 35–50% [26]. This observation reassures that grass is the major component in paunch, where buffel grass is a relevant comparison. The major difference in lignin content between paunch and buffel grass could be explained by the mixed source of grasses fed to the cattle, which could include grass with a higher lignin content than that of buffel grass. When comparing between paunch and buffel grass, it is obvious that paunch had a higher fibrous NDF content. This implies that the digestive process of the cattle could potentially refine the fibrous content naturally and remove the ‘others’ components in the grass, including plant pectin, proteins and lipids. 

**Figure 2:**
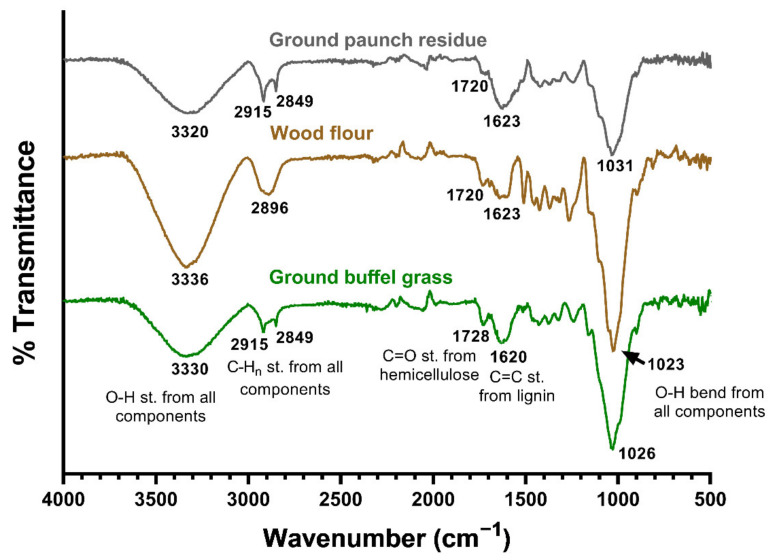
FT-IR spectra of ground paunch, wood flour and ground buffel grass.

FT-IR analysis was used to characterise the surface functional group of the fibres. The functional groups associated with cellulose, hemicellulose and lignin are observed from the spectrum of ground paunch. A very similar FT-IR spectrum was also observed from the literature on clean raw fibres from Aristida adscensionis grass [27], thereby further confirming the grass-like chemistry of paunch. The FT-IR spectra of ground paunch, wood flour and ground buffel grass are shown in Figure 2 for direct comparison. The peaks at 3330 cm^−1^ and 1030 cm^−1^ are linked to the O−H stretching and bending from all three components, respectively. The peaks at 1620 cm^−1^ are assigned to the C=C stretching from the aromatic skeleton in lignin. The peaks at 1720 cm^−1^ correspond to the C=O stretching from the ketone and carbonyl groups in hemicellulose. The major differences of the signature peaks between the three spectra are seen in the region of 2800–2950 cm^−1^. Both the spectra of ground paunch and ground buffel grass have two distinct peaks at 2850 cm^−1^ and 2915 cm^−1^ which can be associated with the C−H stretching from alkyl and aliphatic groups. Only a broad peak was seen in the spectrum of wood flour, the two distinct peaks could be masked by the C−H stretching from aromatics in the lignin components. This supports the results from the compositional analysis above that paunch is more similar to grass than wood. 

### 3.2. Fibre Morphology and Dimensions

**Figure 3:**
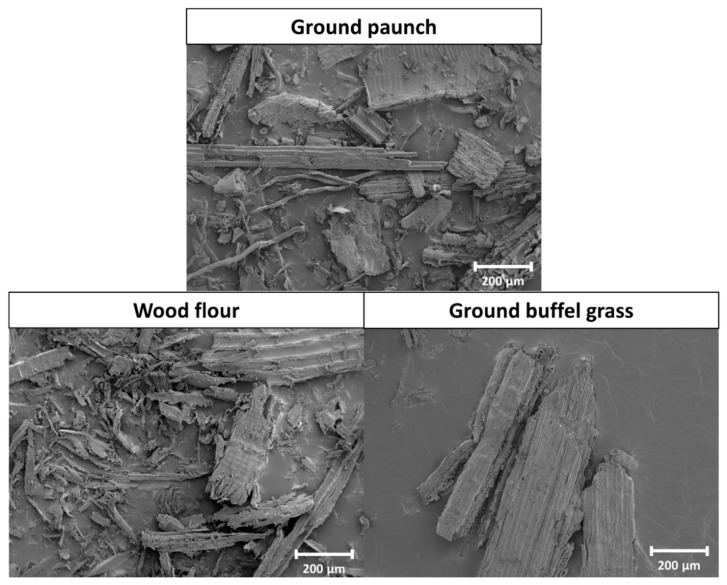
SEM images of ground paunch (**top**), wood flour (**bottom left**) and ground buffel grass (**bottom right**) imaged at 5.00 kV, 10 mm WD and ×100 magnification.

SEM analysis was used to visualise the morphology of ground paunch in comparison to the benchmark single-sourced fibres (wood flour and ground buffel grass). Figure 3 shows the SEM micrographs of ground paunch, wood flour and ground buffel grass. As can be seen from the SEM micrographs in Figure 3, the ground paunch consisted of fibres of different morphologies, including long fibres and thin fibrils. It also contained some non-fibrous materials in large particulate form. These large particulates are suspected to introduce defect points that would initiate crack propagation when under stress. On the other hand, the thin fibrils, which were only observed in the ground paunch, could potentially act as a reinforcement for composites, as high-aspect-ratio fibres have been shown to help distribute the applied load resulting in higher mechanical strength [28,29]. Due to the as-observed wide and random distribution of different materials and fibres in ground paunch, the balance between this reinforcing effect and the density of defect points within the ground paunch composite matrix is the key for achieving mechanical performance comparable to commercial composite products. 

When benchmarking the fibres from paunch against wood flour and ground buffel grass, some similarities were observed. First, the majority of the solids from ground paunch are in fibre-like forms of high aspect ratio, with only a small amount in particulate form. Second, the ridged rough surface of the larger fibres in ground paunch was similar to that observed in wood flour and ground buffel grass, suggesting the presence of undamaged lignocellulosic fibres in the ground paunch. These features hint at the potential of paunch as a valuable fibre source for polymer composites. 

**Figure 4:**
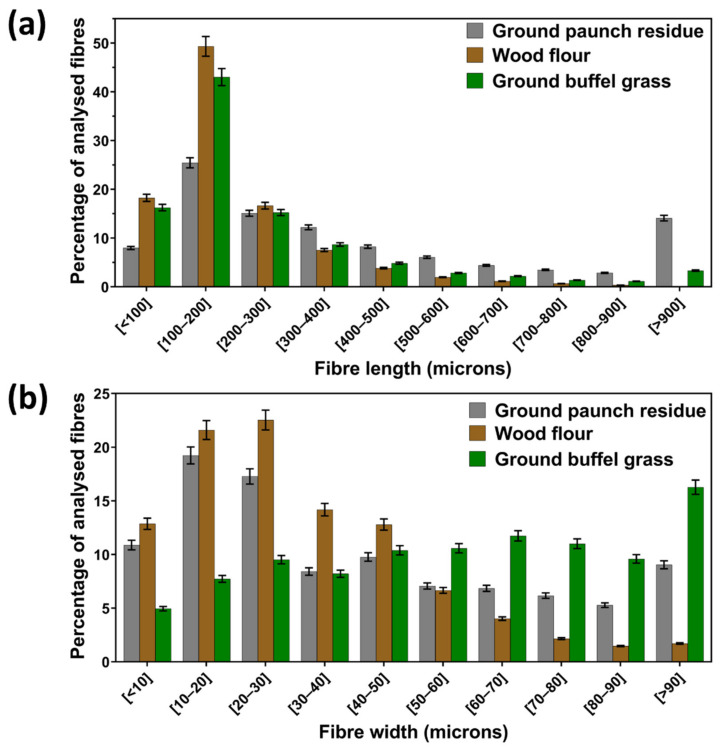
Histograms of the distribution of (**a**) fibre length and (**b**) fibre width of ground paunch (grey), wood flour (brown) and ground buffel grass (green). Average number of fibres analysed: ground paunch = 4100 ± 100, wood flour = 13,100 ± 500 and ground buffel grass = 4200 ± 100. Error bars represent 95% confidence intervals (*n* = 3).

A Morfi fibre analyser was used to quantify the distribution of fibre length and width of the ground paunch as presented in Figure 4, with wood flour and ground buffel grass as references. As can be seen from the grey bar far right in Figure 4a, 14% of the fibres from paunch had a fibre length longer than 900 µm. The average fibre length and width were 235 µm and 41 µm, respectively, resulting in an average aspect ratio of 5.7. This is higher than that of wood flour (aspect ratio: 4.7) and of ground buffel grass (aspect ratio: 4.4). 

### 3.3. Mechanical Properties: Effect of Paunch Loading

**Figure 5:**
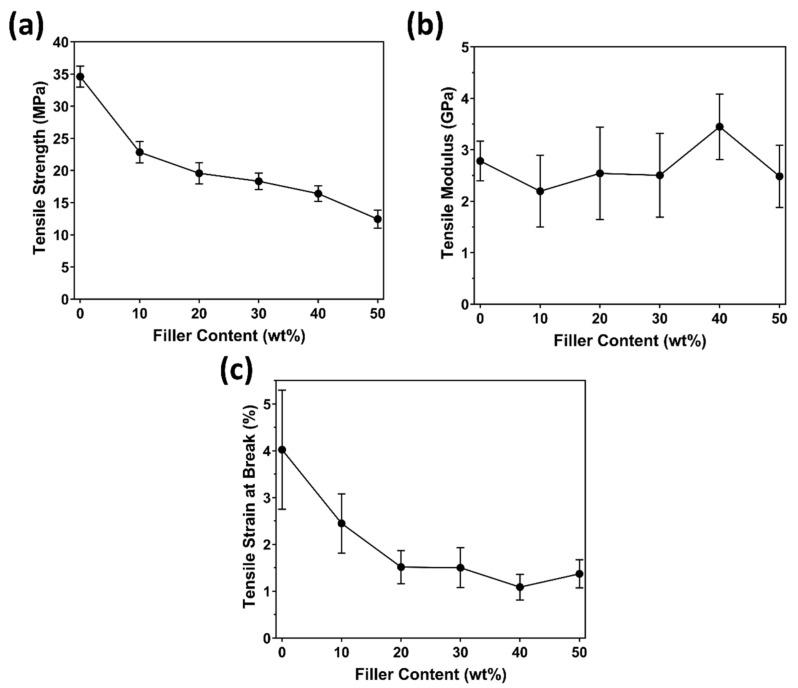
(**a**) Tensile strength, (**b**) tensile modulus, and (**c**) tensile strain at break of PHBV/ground paunch composites, at fibre contents from 0 to 50 wt% at increments of 10 wt%.

The extrusion process was successfully used for producing mechanically intact composites with a ground paunch content of up to 50 wt% without any processing aids, additives and/or pre-treatments. The tensile strength, modulus and strain at break of PHBV/ground paunch composites with increasing fibre content are presented in Figure 5a, b and c, respectively. As can be seen in Figure 5a, the tensile strength decreased with increasing loading of ground paunch. Similar trends have been observed in other composite systems with clean fibres such as wood-based fibres, both with and without compatibilisation [19]. This observation has been associated with the incompatibility between the hydrophobic polymer and the hydrophilic fibre [19]. A higher paunch content results in an increase in interfacial area and thus less polymer relative to surface, potentially leading to weak interactions between wood and polymer and consequently leads to failure at lower loadings.

An increase in ground paunch content also led to a gradual decrease in strain at break, with a close-to linear relationship being observed from 0 to 20 wt% then unchanged. Tensile strain at break is often considered to be the most sensitive mechanical property to voids and loose packing. At higher ground paunch contents (>30 wt%), there is a larger area of imperfect fibre–matrix interface (as indicated in SEM micrographs in the later session). It then could introduce higher density of initial defects and could therefore lead to rapid crack propagation and brittle failure when under stress. 

An improvement in stiffness has been commonly observed in lignocellulosic fibre polymer composites when compared to the raw polymer. However, this is not observed in this PHBV/ground paunch system; the modulus was maintained, but there were no significant increases with increasing paunch content. Overall, the reinforcing effect was not as good as other natural fibres, where ground paunch acted more as a filler. 

### 3.4. Thermal Properties: Effect of Paunch Loading

**Figure 6:**
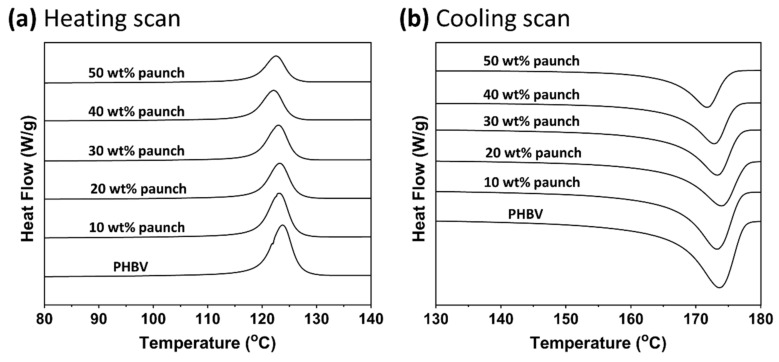
(**a**) First heating scan, and (**b**) first cooling scan from DSC for PHBV in PHBV/ground paunch composites, at fibre contents from 0 to 50 wt%, at increments of 10 wt%.

Figure 6 shows the DSC thermograms of the heating and cooling scans of the as-produced ground paunch composites, with properties as summarised in Table 2. The first heating scan was used to extract information of the PHA crystals properties in their composite product form without eliminating the thermal processing history to access the impact of the ground paunch on polymer properties during processing. As can be seen in Figure 6a, unimodal melting behaviour was observed from all ground paunch composites. This implies that the PHA crystals forms are relatively homogeneous with a single distribution across the composite matrix. In other words, the ground paunch did not cause significant localised disruption of the crystallisation of PHBV. No trends were observed from the enthalpy changes during melting ($\Delta H_{m}$) and melt crystallisation ($\Delta H_{mc}$) with increasing paunch content, suggesting that the bulk crystallinity of PHBV was not affected [30]. This agrees with the observed peak melting and melt crystallisation temperatures, which were also not affected by ground paunch content. 

The crystallisation rate can be estimated by the difference between the starting and ending temperatures ($\Delta T_{mc}$) of the crystallisation peak [31]. Larger differences mean that a longer time is needed for the completion of crystallisation. As can be seen from Table 2, the rate of crystallisation decreased relative to pure PHBV when ground paunch was added, but this was independent of the content. Since that crystallisation is a process in which molecular chains are rearranged, the presence of paunch fibre increased the resistance of PHBV chain movement within the matrix resulting in slower crystallisation rate, but the overall crystallinity was not changed. This behaviour has been observed in wood plastic composite systems [32].

### 3.5. Thermal Stability through Processing: Effect of Paunch Content

Thermal degradation has been observed when PHAs are processed at high temperatures and with high shear [33]. The molecular weight values of all PHAs before extrusion and after extrusion with different loading of ground paunch were therefore characterised to assess the thermal stability of the materials and the effect of ground paunch content on this thermal stability (Table 3). The number of scissions ($N_{s}\left(t\right)$) and average scission rates ($R_{s}$) were also estimated using Equations (1) and (2) [34] and are presented in Table 3:

(1) $$N_{s}\left(t\right)=\frac{\bar{M_{w}}\left(t=0\right)}{\bar{M_{w}}\left(t\right)}-1$$

(2) $$R_{s}=\frac{N_{s}\left(t\right)}{residence\;time\;in\;extruder=1\;min}$$

The molecular weight of PHBV dropped after extrusion processing and this decrease is more severe when ground paunch is added to the formulation. Molecular weight is an important parameter to determine polymer properties, since longer chains lead to better mechanical performance, at least within the typical range of molecular weights. The PHBV matrix has been shown to retain mechanical strength until the ${\bar{M}}_{w}$ has decreased to lower than 134 kDa [35]. At 50 wt% paunch, the ${\bar{M}}_{w}$ of PHBV was 223 kDa, which is higher than the threshold for mechanical loss based on literature data. 

The average scission rate increased gradually with increasing ground paunch content. Similar trends have also been reported for PHBV/bleached kraft fibre composites [36]. The PHBV has a higher chance of experiencing high shear when processed with ground paunch due to their rough fibre surface. As described earlier with evidence from the SEM image in Figure 3, an uneven surface was observed from the larger fibres in the ground paunch sample. During extrusion processing, the PHBV was rubbed against the ridged fibre surface that could generate localised shear and heat due to friction. Such heat shear processing could result in an increased degree of thermal degradation [37]. Furthermore, the presence of moisture in the ground paunch could also facilitate the hydrolytic chain scissioning of the PHA and potentially caused more severe loss of molecular weight [38]. Although the ground paunch was dried before processing, there were still unavoidable exposure to surrounding moisture during feeding due to an imperfect off-line dryer-extruder system. 

The polydispersity index (PDI), which is a measure of the heterogeneity of the length of the polymer chains, was calculated for each sample and is shown in Table 3. A decreasing trend was observed with increasing paunch content, i.e., the thermal degradation narrowed down the molecular weight distribution of PHBV in the composite matrix. 

### 3.6. Mechanical Properties: Comparison across Different Fibres

**Figure 7:**
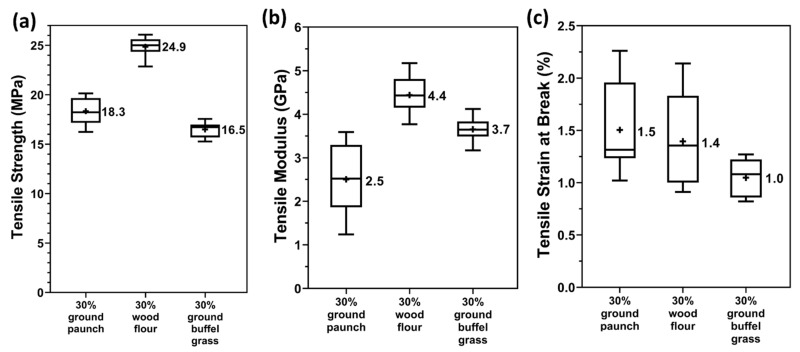
(**a**) Tensile strength, (**b**) tensile modulus, and (**c**) tensile strain at break of PHBV/ground paunch, PHBV/wood flour and PHBV/ground buffel grass composites at a fibre content of 30 wt%.

In order to assess the potential of paunch in composite applications, the mechanical properties of PHBV/ground paunch composites with 30 wt% (representing a balance between cost-competitiveness and mechanical performance) were benchmarked against PHBV/wood flour and PHBV/ground buffel grass composites. The tensile properties of the composites at 30 wt% are presented as box-and-whisker plots, with mean values on the side, in Figure 7. In general, the mechanical properties of PHBV/ground paunch composites were more like those of ground buffel grass. This is expected, as they are of similar origin, with grass being an animal feed. Composites with ground paunch had a tensile strength 10% higher but a modulus 50% lower than that of ground buffel grass composites. Ground paunch provided less stiffness improvement to PHBV than ground buffel grass but the smaller fibre size (235 µm length and 41 µm width for ground paunch compared to 258 µm length and 58 µm width for ground buffel grass) limited the defect points within the composite matrix, yielding a material that can sustain higher loading. By contrast, composites with ground paunch yielded tensile strength and modulus significantly lower than that of wood flour (by 36% and 76%, respectively). Wood exhibits high stiffness and has been widely established as a reinforcing fibre for commercial polymer composites and thereby serves as a high benchmark. Still, it is not necessary to match the properties of wood–PHA composites as some applications require less strength but lower cost. The tensile strains at break for all composites were similar and fall into the brittle failure category, which is expected for PHBV composites [19].

### 3.7. Interfacial Interactions: Comparison across Different Fibres

**Figure 8:**
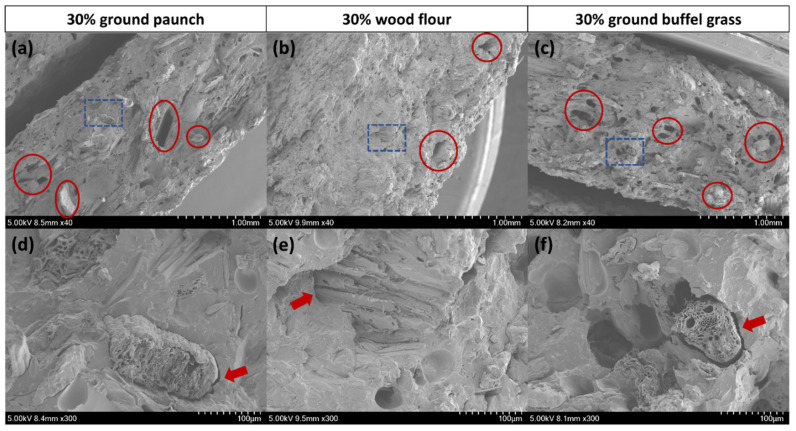
SEM micrographs of the fractured surface of (**a**,**d**) PHBV/ground paunch, (**b**,**e**) PHBV/wood flour and (**c**,**f**) PHBV/ground buffel grass composites at a fibre content of 30 wt%. The magnified regions are indicated by blue-dashed boxes. Key features are annotated by red circles for fibre pull-outs and red arrows for interface regions.

In addition to the fibre properties, the level of fibre–matrix interfacial adhesion is a key property in determining composite mechanical properties [39]. The increased number of voids and the presence of fibre pull-outs from the SEM micrographs of fractured surfaces have been established as an indication of worsened interfacial adhesion resulting in the detachment of the fibres rather than breakage [40,41,42]. Cavities and detached fibre without breakage are evident in all composites which indicated imperfect interfacial adhesion (red circles in Figure 8a–c). When comparing across different fibres, higher density of cavities and detached fibre were observed from PHBV/ground paunch and PHBV/ground buffel grass than that of PHBV/wood flour composites. Furthermore, wider spacing between the fibres and surrounding matrix was observed from PHBV/ground paunch and PHBV/ground buffel grass when compared to PHBV/wood flour composites as indicated by red arrows in Figure 8d–f. Both observations are consistent and suggested a relatively limited fibre–matrix interaction with grass, which could explain the observed better mechanical properties from the composites with wood flour. 

### 3.8. Thermal Properties and Thermal Stability: Comparison across Different Fibres

Thermal properties showed that the PHBV in PHBV/ground paunch composites exhibited similar melting and crystallisation profiles to that in wood flour and ground buffel grass composites (Table 4). Similar melting temperature (Peak $T_{m}$), melt crystallisation temperature (Peak $T_{mc}$) and crystallisation rate ($\Delta T_{mc}$) are key evidence. Lower enthalpy of melting (Δ*H_m_*) values were observed from PHBV/ground buffel grass composites, where ground paunch and wood flour composites had similar values. The data suggested that processing of ground paunch composites resulted in a similar PHBV matrix material to that when processed with wood flour. Among the three fibres, only the presence of ground buffel grass suppressed the bulk crystallinity of PHBV [30]. Buffel grass contains a larger amount of non-neutral detergent fibre content (Table 1) than paunch and wood flour. The presence of these ‘other’ materials may have disturbed the spherulite growth of PHBV crystals leading to a lower bulk crystallinity. The lower crystallinity of PHBV in PHBV/ground buffel grass composites could contribute towards their observed lower tensile strength [43].

At 30 wt% fibre loading, the thermal degradation of PHBV when processed with ground paunch were similar to that of wood flour and ground buffel grass (Table 5). This indicated that, despite the unrefined nature, ground paunch did not pose any detrimental effect in composite processing demonstrating its potential as a fibre source. 

## 4. Conclusions

Composites of PHBV and ground paunch procured from the waste streams in a red meat processing facility were successfully produced via extrusion, with up to 50 wt% fibre content and without any pre-treatment and compatibilisation. This paper presents the first study, to our knowledge, on exploring the fibre properties of a paunch sample and the resulting properties of its composites. This paunch had a rough surface morphology and fibre-like characteristics which shared similar fibre morphology and wood chemistry to virgin ground buffel grass. Paunch had a higher content of structural components (cellulose, hemicellulose, and lignin) than buffel grass that is comparable to wood flour, demonstrating the unique advantage of paunch on the natural refining process through the digestive process of the cattle. 

Mechanical testing, thermal analysis and molecular weight determination showed that the property trends upon the addition of increased loadings of ground paunch to the PHBV matrix behaved very similar to those of other filler-filled composites. Mechanical results showed that the reinforcing effect is not as good as other fibres, where the ground paunch acted more as a filler. At 30 wt% content, PHBV/ground paunch composites yielded mechanical properties comparable to composites with ground buffel grass, although they were still lower than those of composites with wood flour. In summary, without any further refining and pre-treatments, the paunch waste from red meat processing facilities has demonstrated its potential as a valuable fibre source to be used as a filler for polymer composites. Future research is directed to investigate paunch fibre refinement strategies with the aim being to further enhance the mechanical properties of paunch composites for wider engineering applications.

## Figures and Tables

**Table 1:** Cellulose, hemicellulose and lignin contents of ground paunch, wood flour and ground buffel grass.

	Ground Paunch	Wood Flour	Ground Buffel Grass
Neutral Detergent Fibre (NDF)	86.9%	90.6%	69.6%
Cellulose	38.6%	41.2%	36.0%
Hemicellulose	32.6%	17.4%	29.0%
Lignin	15.6%	32.0%	4.6%

**Table 2:** Thermal properties for PHBV in PHBV/ground paunch composites, at fibre contents from 0 to 50 wt%, at increments of 10 wt%.

	Melting Behaviour		Crystallisation Behaviour		
	Peak *T_m_* (°C)	Δ*H_m_* (J/g-PHA)	Peak *T_mc_* (°C)	Δ*T_mc_* (°C)	Δ*H_mc_* (J/g-PHA)
PHBV	174	94.2	124	33	88.9
10% ground paunch	173	91.0	123	43	91.2
20% ground paunch	174	82.1	123	42	83.9
30% ground paunch	173	87.1	123	40	89.0
40% ground paunch	173	83.8	122	44	91.0
50% ground paunch	172	88.6	122	40	97.0

**Table 3:** Molecular weight values of PHBV before and after extrusion and PHBV/ground paunch composites after extrusion at fibre contents from 10 to 50 wt%, at increments of 10 wt%.

	${\bar{M}}_{n}\;(\mathrm{kDa})$	${\bar{M}}_{w}\;(\mathrm{kDa})$	$R_{s}\;(\min^{-1})$	PDI
As-received PHBV powder	202	532	--	2.64
Extruded PHBV	177	395	0.35	2.23
10% ground paunch	168	391	0.36	2.33
20% ground paunch	161	355	0.50	2.20
30% ground paunch	139	306	0.74	2.21
40% ground paunch	132	279	0.91	2.11
50% ground paunch	107	223	1.39	2.08

**Table 4:** Thermal properties for PHBV in PHBV/paunch, PHBV/wood flour and PHBV/buffel grass composites at a fibre content of 30 wt%.

	Melting Behaviour		Crystallisation Behaviour		
	Peak *T_m_* (°C)	Δ*H_m_* (J/g-PHA)	Peak *T_mc_* (°C)	Δ*T_mc_* (°C)	Δ*H_mc_* (J/g-PHA)
30% ground paunch	173	87.1	123	40	89.0
30% wood flour	174	84.8	120	39	87.9
30% ground buffel grass	174	68.1	118	43	69.2

**Table 5:** Molecular weight values of PHBV in PHBV/ground paunch, PHBV/wood flour and PHBV/ground buffel grass composites at a fibre content of 30 wt%.

	${\bar{M}}_{n}\;(\mathrm{kDa})$	${\bar{M}}_{w}\;(\mathrm{kDa})$	$R_{s}\;(\min^{-1})$	PDI
30% ground paunch	139	306	0.74	2.21
30% wood flour	138	312	0.71	2.26
30% ground buffel grass	140	328	0.62	2.41

## Data Availability

The data presented in this study are available on request from the corresponding author.
